# Role of Platelet-Rich Plasma in Spinal Fusion Surgery: Systematic Review and Meta-Analysis

**DOI:** 10.1155/2020/8361798

**Published:** 2020-05-06

**Authors:** Daudi R. Manini, Frank D. Shega, ChaoFeng Guo, YuXiang Wang

**Affiliations:** ^1^Department of Spine Surgery, Xiangya Hospital of Central South University, Changsha 410008, Hunan Province, China; ^2^Department of Orthopedic Surgery, Mwananyamala Regional Referral Hospital, P.O. Box 61665, Dar-es-Salaam, Tanzania; ^3^Department of Orthopedic Surgery, Mount Meru Regional Referral Hospital, P.O. Box 3092, Arusha, Tanzania

## Abstract

**Background:**

Platelet-rich plasma (PRP) has been used very successfully in enhancing bone fusion in animal experiments. Also, the efficaciousness of PRP in other specialties of medicine such as dentistry, dermatology ophthalmology, and sports medicine is well documented. But the use of PRP to augment bone fusion after spinal surgery in humans is still controversial. We conducted a meta-analysis to determine the role of PRP in enhancing spinal fusion by fastening the rate of new bone formation and decreasing pain after spinal surgery in humans.

**Methods:**

We searched PubMed, EMBASE, and the Cochrane Library studies that compared PRP versus control in enhancing spinal fusion after deformity correction.

**Results:**

Five retrospective studies with 253 participants and nine prospective cohort studies with 460 participants were identified. The bone fusion rate was excellent for studies that used a high platelet concentration in PRP relative to control (odds ratio (OR) = 4.35, 95% confidence interval (CI) (2.13, 8.83), and *P* < 0.05) while bone fusion was poor to studies that used a low concentrate of platelet in PRP relative to control. The rate of new bone formation was high in the PRP group compared to the control group with the mean difference in Hounsfield unit (HU) 144.91 (95% CI (80.63, 209.18), *P* < 0.05). Time to bone fusion was short in the PRP group during the first six months of surgery relative to the control group with a mean difference of −2.03 (95% CI (−2.35, −1.7); *P* < 0.05). No difference was found in pain reduction by visual analog score (VAS) between the PRP group and control.

**Conclusion:**

PRP facilitates new bone formation and bone fusion with a minimum concentration of the growth factor 5 times that of the peripheral blood. PRP stimulatory effects are not continuous and are very effective within six months of implantation.

## 1. Introduction

Platelet-rich plasma (PRP) is blood plasma with concentrated autologous platelets and various growth factors as well as cytokines above the normal baseline level. PRP is believed to contain several growth factors including platelet-derived growth factor (PDGF), transforming growth factor (TGF), insulin-like growth factor (IGF), epidermal growth factor (EGF), epithelial cell growth factor (EGR), and hepatocyte growth factor (HGF). These growth factors can promote the healing of bone and soft tissues [1–3]. Spinal fusion is a commonly performed procedure in the treatment of spinal instability due to different spinal pathologies. Despite the current advanced techniques in spinal fusion, failure of fusion and pseudarthrosis are still the main challenges. Therefore, additional materials such as bone graft extenders and biologics such as PRP are employed during spinal fusion surgery to enhance fusion [4, 5].

The use of PRP has been very successful in enhancing spinal fusion in an animal model [6, 7]. Also, PRP has been broadly and efficaciously used in other specialties of medicine such as oral dentistry, dermatology, ophthalmology, and sports medicine [8]. But the use of PRP to augment bone fusion after spinal deformity correction in humans is still controversial; Kubota et al. [9] in their prospective randomized control trial of posterolateral lumbar fusion surgery found a high fusion rate in the PRP group compared to control. Tarantino et al. [10] in their prospective cohort study of 21 patients who underwent posterolateral arthrodesis with implantation of cancellous bone substitute soaked with PRP found that PRP increases the rate of fusion and bone density adding osteoinductive and osteoconductive effect. Also, Hartmann et al. [11] in their 15 controlled cohort patients who underwent anterior spinal fusion after suffering lumbar or spinal injury found that PRP increased the rate of fusion and high-density value within the region of fusion compared to control. On the other hand, Feiz-Erfan et al. [12] in their double-blind randomized study with platelet-gel concentrate or control group of 50 patients undergoing anterior cervical microdiscectomy, allograft fusion, and plating found that platelet-gel concentrate had no consistent effect in promoting early fusion in cervical disc disease relative to control. Carreon et al. [13] in their retrospective cohort study of 76 patients who underwent posterolateral lumbar fusion with autologous iliac crest bone graft mixed with autologous growth factor (AGF) and control found a high nonunion rate in the AGF group compared to control. Also, Jenis et al. [14] in their prospective study of patients undergoing lumbar spinal fusion using iliac crest autograft and allograft combined with autogenous growth factors (AGF) found a similar outcome in terms of bone fusion, pain, and functional improvement between the two groups.

Therefore, the aim of this study is to investigate the role of PRP in promoting bone fusion after spinal surgery in humans, by using available published studies through meta-analysis.

## 2. Methods

### 2.1. Eligibility Criteria

Included articles had the following inclusion criteria: (1) studies conducted in humans evaluating spinal fusion; (2) studies comparing the outcome between PRP and control subject; (3) studies evaluating spinal fusion by either radiograph or computed tomography (CT); (4) the sample size was bigger than 10; (5) only retrospective/prospective studies published in English; and (6) no geographic restriction was set.

Exclusion criteria are as follows: (1) case reports and review studies; (2) studies based on animal research or cadaveric research; (3) studies that did not evaluate spinal fusion; and (4) studies that did have control subjects.

### 2.2. Information Sources

Three online databases, PubMed, EMBASE, and the Cochrane Library, were searched to come up with the list of eligible included studies. No date range customization of searches was set. Corresponding authors of articles searched could be contacted to provide further information or settle unclear explanations. Secondary referencing of eligible studies was done to extend the scope of the searches. The online databases were accessed via the Central South University Library's website: http://lib.csu.edu.cn/.

### 2.3. Study Search

To generate a set of citations that are relevant to our study's search question, an advanced search tool was used in all of the three databases previously mentioned. Free text words, as well as MeSH terms, were used to search. Using PubMed, advanced search builder was customized to “Title,” “*human species*,” and different combinations of free text words were run for search: “*cervical vertebrae*,” “*thoracic vertebrae*,” “*lumbar vertebrae,*” “*platelet-rich plasma,*” and “*spinal fusion”* connected by “AND.” The searches were independently performed by two authors; differences of thought were settled through discussion with the third author. Results were exported to computer software, EndNote X9 (Bld 12062), which was used to manage and keep track of references throughout the study.

### 2.4. Study Selection Process

**Figure 1:**
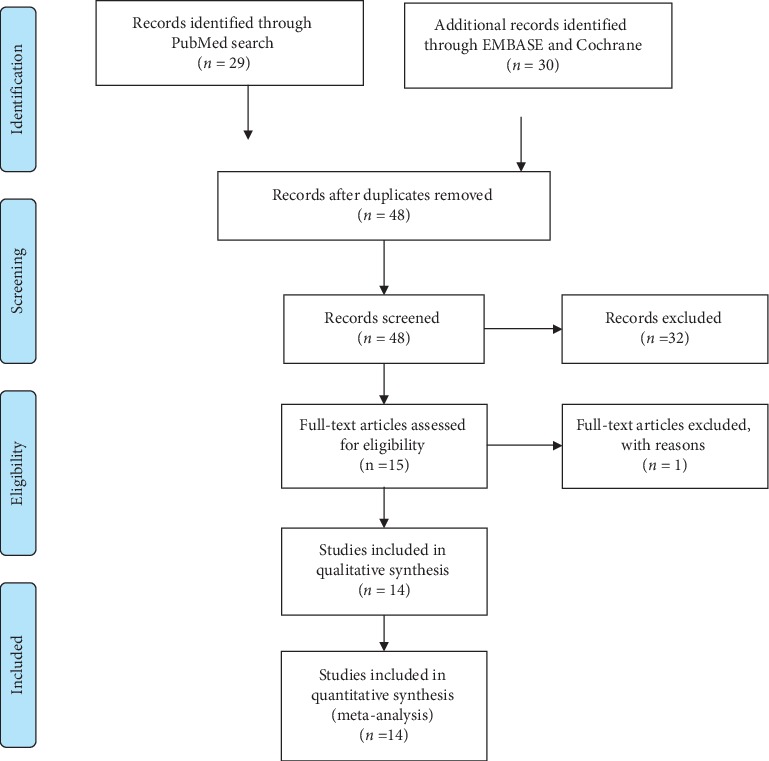
Study selection process according to PRISMA guidelines.

All studies resulting from online database search independently conducted by two authors were screened by their titles and abstracts to initially assess their relevance to our study question and grossly irrelevant articles were discarded. This was level-one screening and was done by the same two authors: Daudi Manini and Frank Shega. Compiled search results of level-one screening were then searched for their full-text articles from which eligibility for inclusion or exclusion was sought. Any differences of thoughts in the search process were settled by Wang YuXiang. The study search and selection process are summarized in Figure 1.

### 2.5. Data Extraction

Data from included articles were extracted by Daudi and Frank independently. Disagreements were solved through discussion with Wangyuxiang. Data extracted from each of the included studies were as follows: study title, author, and year of publication, the number of participants, study design, the region of spinal fusion, the material used for bone fusion, follow-up time, and tools used to assess bone fusion between PRP and control group; the number of bone fusion and the time to bone fusion between PRP and control; and means and standard deviation of the newly formed bone within the region of interest (ROI) in terms of density expressed as Hounsfield unit (HU) and visual analog score (VAS) for pain between PRP and control groups.

### 2.6. Quality Assessment

The quality assessment of the 14 included studies was done independently by two authors, and disagreement was solved through discussion with the 3rd author. All included studies scored more than 8 stars on the Newcastle–Ottawa scale (NOS). This means the quality of all included studies was high. The results for the NOS score for each study are summarized in Table 1.

### 2.7. Statistical Analysis

The Review Manager software (RevMan 5.3, The Cochrane Collaboration, Oxford, UK) was used to perform statistical analysis. Continuous variables were reported as mean difference (MD) and 95% confidence interval (95% CI), while dichotomous data were presented as odds ratio (OR) and 95% CI. A random-effects model was used for heterogeneous data (*I*^2^ ≥ 50% for heterogeneity test). Collected data were entered into the computer and rechecked by two authors.

Some articles did not provide the change values and their standard deviation (SD) from preoperation to postoperation, but reported data before and after surgery, respectively. To get the data we needed, we consulted the authors for the data. And in articles that gave raw data, we computed the mean and standard deviation by IBM SPSS statistic 21 version software.

### 2.8. Assumptions and Simplifications

All participants, despite study countries' economic status and technological differences, were considered to have received standard surgical care.

## 3. Results

### 3.1. Study Selection

Eligible included studies were prospective and retrospective cohort comparing mean differences between PRP and control by measuring the rate of new bone formation in terms of HU and pain reduction in terms of VAS and also, the number of bone fusion and the time to bone fusion in terms of OR between the two groups at the final follow-up.

A total of 59 studies were obtained from 3 online databases. Study search from PubMed resulted in 29 studies, EMBASE resulted in 22 studies, and COCHRANE resulted in 8 studies. After the removal of 11 studies which were duplicates, 48 studies were identified for screening. After reading abstract and title, 32 studies were irrelevant, and thus, 15 studies were sought for their full texts. After reading full texts of 15 studies, 1 study did not meet the eligibility criteria for our study. 14 studies were selected and included in our meta-analysis. The characteristics of the included studies are summarized in Table 2.

### 3.2. Study Bias

Bias in the included studies was assessed by NOS. NOS is approved by Cochrane Handbook version 5.1 to assess bias in observational studies.

In terms of study design, 5 studies were retrospective cohort [13, 15, 17, 19, 21] and 9 studies [2, 9, 11, 12, 14, 16–18, 20] were prospective. Generally, prospective studies have less bias compared to retrospective studies. The sample size from all included studies was small and none of them calculated the sample size before the conduction of the study. The smaller the sample size, the lesser the representative of the general population. Different techniques and fusion materials were used in spinal fusion surgery. Some surgeons employed PLF, TLIF, or IF as shown in Table 2.

### 3.3. Bone Fusion

**Figure 2:**
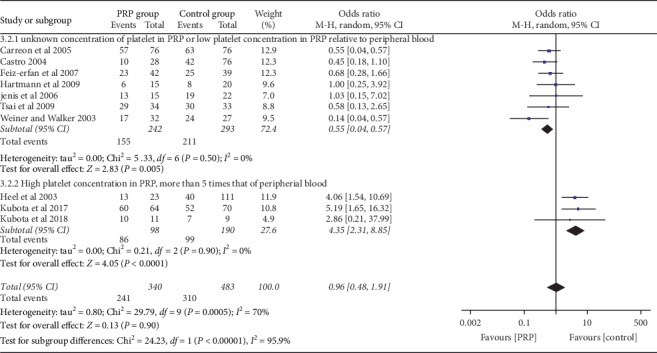
Forest plot for bone fusion between the PRP and control groups at the final follow-up.

The data for bone fusion were available in 10 studies as shown in Figure 2 from which one study [19] used a low platelet concentration in PRP (3.5 higher than the peripheral blood) and the 6 studies did not report the platelet concentration in their PRP [11–14, 18, 21]. The bone fusion in these seven studies was poor in the PRP group compared to control (odds ratio (OR): 0.55 at 95% CI (0.36, 0.83) and *P*=0.005). Three studies [9, 15, 20] used a high platelet concentration in the PRP, 5 times higher than peripheral blood. The rate of bone fusion in the PRP group was excellent relative to the control group (OR = 4.35, 95% CI (2.15, 8.85), and *P*=0.0001).

But the total effect of PRP on bone fusion in all 10 studies was poor compared to the control group (OR = 0.96, 95% CI (0.48, 1.96), and *I*^2^ = 95.9) with high heterogeneity.

### 3.4. The Density of the Newly Formed Bone in ROI

**Figure 3:**
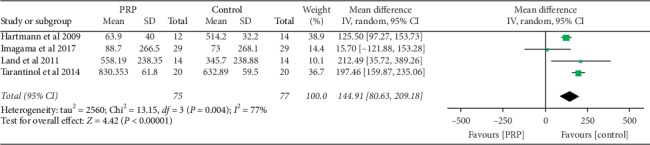
Forest plot for analysis of the newly formed bone in (HU) in ROI between the PRP and control groups.

The data for the density of the newly formed bone in ROI in HU between PRP and control groups were available in 4 studies [2, 10, 11, 17] as shown in Figure 3. The rate of newly formed bone mass was high in the PRP group compared to the control group, and the results are statistically significant with the mean difference of 144.91 (95% CI (80.63–209.18), *I*^2^ = 77%, and *P* < 0.05)

### 3.5. Time to Bone Fusion

**Figure 4:**
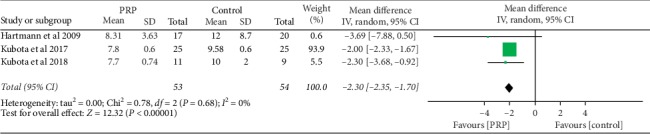
Forest plot for time to bone fusion in months between the PRP and control groups.

The data for time to the bone fusion between PRP and control groups were available in 3 studies [9, 11, 15] as shown in Figure 4. There was less time for a bone fusion in the PRP group compared to control. The results are statistically significant with a mean difference of −2.05 (95% CI (−2.35–1.70); *P* < 0.05).

### 3.6. Pain

**Figure 5:**
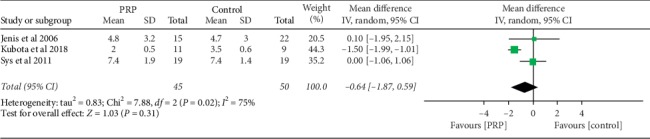
Forest plot for analysis of pain at the final follow-up in terms of VAS between the PRP and control groups.

The data for the analysis of pain in terms of VAS were available in 3 studies [14–16] as shown in Figure 5. There was no statistical difference in pain reduction between the PRP and the control groups at the final follow-up. The mean VAS score was −0.64 (95% CI (−1.87–0.59), *I*^2^ = 75%, and *P*=0.31).

## 4. Discussion

The use of PRP has been very successful in enhancing spinal fusion in an animal model [6, 7]. Also, PRP has been broadly and efficaciously used in other specialties of medicine such as oral dentistry, dermatology, ophthalmology, and sports medicine [8]. But the use of PRP to augment bone fusion after spinal deformity correction in humans is still controversial. Some scholars advocate the use of PRP to facilitate bone fusion during spinal surgery while other scholars are against the use of PRP to facilitate spinal fusion.

In our meta-analysis, we found that PRP promotes bone fusion when the concentration of platelet is very high. All researchers [2, 9, 15, 20], who used high platelet concentration in PRP, 5 times higher in the peripheral blood, found superior bone fusion in PRP compared to control. But the researcher in [19] who used a low platelet concentration in PRP found no difference in bone fusion rate between the PRP and control groups. The bone fusion rate in the PRP group was also poor in studies [11, 12, 14, 18, 20, 21] which did not measure the concentration of platelet in PRP relative to that of the baseline. Probably, the concentration of platelet in their PRP was not higher than 4- to 5-fold above the peripheral blood which has been reported in the literature to cause a stimulatory effect [5]. Kubota et al. [9] in their prospective study of 31 patients in both PRP and control groups, who underwent posterolateral lumbar fusion with local bone graft and PRP or local bone graft alone, used platelet concentration 7.7 times high in PRP and growth factor concentration 50 times higher than that of the plasma, and they found excellent bone fusion in PRP relative to the control group. Both the positive and negative effects of PRP in promoting bone fusion in animal and clinical research have been reported. Furthermore, it was argued that PRP had stimulating effects if the concentration of the platelet counts and that of the growth factor in PRP are extremely higher than those in the peripheral blood. Also, PRP does not continue to provide stimulatory effects for a long period of time. Hence, the amount and concentration of platelet in PRP in large-sized animals like humans being or to the tissue that takes a long time to regenerate like bone must be increased [22, 23]. This hypothesis has been proved in this meta-analysis.

In this study, we found that PRP is superior in promoting new bone formation compared to control. The data of new bone formation in the ROI were available in four studies [2, 10, 11, 17]. There was a statistically significant difference between the two groups (*P* < 0.05). Heterogeneity was high probably because of the different surgical techniques and material used during surgical fusion. This result is similar to what has been reported by other scholars. Imagama et al. [2] in their prospective clinical study of 29 patients in both experimental and control, who underwent PLF with both local bone and PRP in experimental and local bone only in control, found that the rate of bone formation in control was higher in the experiment group at 3 and 6 months of follow-up than control. The authors found no differences in bone formation between the two groups during the 12 months of follow-up. Also, in Hee et al.'s retrospective study [20], 23 patients who underwent transforaminal lumbar interbody fusion (TLIF) with AGF and autograft were compared with 111 control patients, who underwent TLIF with autograft alone. Radiographic assessment for bone fusion was done at 4, 6, and 24 months of follow-up time; more rapid incorporation of bone fusion was observed at 4 and 6 months in AGF patients; and during 24 months of assessment, no significant differences in bone fusion rate were found between the two groups. And the authors concluded that AGF was able to promote graft incorporation, hence speeding up faster fusion.

In this study, we also found that time to bone fusion was short in the PRP group compared to the control group. This finding is similar to what has been reported by other scholars. Tarantino et al. [10] found that PRP fastens bone fusion in PLF surgery by measuring bone density in the fusion area using CT scan on 20 patients who underwent PLF surgery with cancellous bone soaked with PRP and saline on right and left sides, respectively. Also, Imagama et al. [2] observed a fast bone fusion rate at 3 and 6 months after surgery, and authors found no difference in the bone fusion between PRP and control groups during 12 months of follow-up. On long-time follow-up, there was no difference in bone fusion between PRP and control groups, possibly because PRP does not continue to provide stimulatory effect for a long period of time [22, 24].

Our meta-analysis did not find any statistically significant difference in pain score during the final follow-up between the PRP and control groups. This is similar to what has been reported by other scholars. Kubota et al. [15] in their retrospective case series of 20 patients who underwent TLIF with local bone graft with PRP and local bone graft alone in the control group found no significant difference in lower back pain, leg pain, and leg numbness between the two groups at the final follow-up. Also, Sys et al. [16] in their retrospective study of 38 patients who underwent posterior stabilization with autograft addition to PRP and autograft alone in the control group found improvement of VAS in both groups, but the difference was not statistically significant between the groups at the final follow-up.

Our meta-analysis has several limitations, the sample space and the number of the included articles were few, and none of them calculated the sample size prior to the conduction of the study. There was variability among studies with respect to surgical technique, methods of preparation of PRP, concentration of platelet in PRP, and dosage as well as the carrier of PRP. Furthermore, there was variability in terms of follow-up time among the included study. All these reduce the overall quality of its evidence.

The main finding of this meta-analysis is as follows: PRP promotes bone fusion and new bone formation well within 6 months of implantation, and after 6 months, the effect normalizes. Also, a minimum concentration of platelet in PRP, 5 times higher than that in the peripheral blood, has a stimulatory effect on bone fusion. This is clinically significant because it advocates that PRP could be employed to fasten postoperative recovery and hence shorten the time for rehabilitation and the use of orthopedic devices. Also, PRP is less expensive and easy to prepare and has no risk of disease transmission. We suggest future studies to focus much on the stable carrier of PRP which allows the continuous release of growth factor to the local tissue for a long period, optimal implantation time, frequency of implantation, and bioavailability of the growth factors.

## 5. Conclusion

PRP facilitates new bone formation and bone fusion with a minimum concentration of the growth factor 5 times that of the peripheral blood. PRP stimulatory effects are well effective within 6 months of implantation.

## Figures and Tables

**Table 1:** Quality assessment of the included studies according to the Newcastle–Ottawa scale.

References	Selection	Comparability	Outcome	Total
Kubota et al. [15]	3	2	4	9
Kubota et al. [9]	3	2	4	9
Tarantino et al. [10]	3	1	4	8
Sys et al. [16]	3	1	4	8
Land et al. [17]	4	1	4	9
Tsai et al. [18]	4	1	3	8
Hartmann et al. [11]	4	2	3	9
Feiz-Erfan et al. [12]	4	2	3	9
Jenis et al. [14]	4	1	3	8
Carreon et al. [13]	4	2	3	9
Castro[19]	4	2	3	9
Hee et al. [20]	4	2	3	9
Weiner and walker [21]	4	2	3	9
Imagama et al. [2]	4	2	3	9

**Table 2:** Characteristics of included studies.

Studies	Study design	Fusion type	Conc of PLT in PRP	Preparation of PRP	Material used	Sex (m/f)	Follow-up time (months)	Evaluation
1. Kubota et al. [15]	Retrospective case series	Lumbar PLF	8.7	400 mL of peripheral blood centrifuged. Buffy coat isolated. The second centrifugation done. 22 mL mixed with calcium chloride.	Local bone, CA, PRP (*n* = 11) Local bone CA (*n* = 9)	10/10	24	CT

2. Kubota et al. [9]	Prospective	Lumbar PLF	7.7	400 mL of peripheral blood centrifuged. Buffy-coat isolated. The second centrifugation done. 22 mL mixed with calcium chloride.	ABG lamina, PRP (*n* = 25) ABG lamina (*n* = 25)	29/21	24	Radiograph, CT

3.Tarantino et al. [10]	Prospective cohort	Lumbar PLF	Not measured	Venous blood centrifuged at 3100 rpm for 8 minutes. Buff coat removed. Platelets were suspended in plasma while shaking the tubes and were ready for use.	Heterologous bone, PRP (*n* = 20) Heterologous bone (*n* = 20)	8/12	6	CT

4. Sys et al. [16]	Prospective	Lumbar IF	Not measured	54 mL of peripheral blood put in SymphonyTM Platelet Concentration System (DePuy, Johnson & Johnson).	CA, ABG iliac, PRP (*n* = 19) CA, ABG iliac (*n* = 19)	24/14	12	Radiograph, CT

5. Land et al. [17]	Retrospective	Thoracic or lumbar IF	5	16 mL of peripheral blood in REGEN-THT_ (thrombocyte harvesting tube) followed by the addition of Ca gluconate and ethanol.	ABG local bone, PRP (*n* = 14) ABG local bone (*n* = 14)	9/5	6	Radiograph CT

6. Tsai et al. [18]	Prospective	Lumbar PLF	Not measured	10 mL of FFP mixed with calcium chloride. Mixture shaken for 30 minutes and ready for use.	ABG lamina PRP (*n* = 33) ABG lamina (*n* = 34)	17/50	24	Radiograph, CT

7. Hartmann et al. [11]	Prospective	Lumbar or thoracic PLF	Not measured	110 mL PVB in Gravitational Platelet Separation (GPS™) System mixed with thrombin.	CA, ABG fracture, PRP (*n* = 15) CA, ABG fracture (*n* = 20)	23/12	8	CT

8. Feiz-Erfan et al. [12]	Prospective	Cervical IF	Not measured	Anticoagulated blood put in Symphony (DePuy, Johnson & Johnson).	Cortical allograft bone, PRP (*n* = 42) Cortical allograft bone alone (*n* = 39)	21/29	24	Radiograph

9. Jenis et al. [14]	Prospective	Lumbar PLF	Not measured	450 mL PVB in centrifuge. Buffy coat mixed with thrombin.	Allograft bone, PRP(*n* = 15) ABG iliac bone (*n* = 22)	24/13	24	Radiographic CT

10. Carreon et al. [13]	Retrospective cohort study	Lumbar PLF	Not measured	500 mL PVB, in a centrifuge. A buffy coat mixed with calcium chloride.	ABG iliac, PRP (*n* = 76) ABG iliac alone (*n* = 76)	40/36	24	Radiographic CT

11. Castro et al. [19]	Retrospective	TLIF	3.5	1 unit of PVB in centrifuge. A buffy coat mixed thrombin	AGF, iliac bone (*n* = 22) iliac bone (*n* = 62)	27/57	41	Radiograph

12. Hee et al. [20]	Prospective	Lumbar PLF	4.89	450 cc of WB in centrifuge buffy coat mixed with thrombin	CA, ABG iliac (*n* = 23) ABG iliac (*n* = 111)	50/84	24	Radiograph

13. Weiner and Walker [21]	Retrospective	Lumbar PLF	Not measured	From 1 unit of arterial blood, buffy coat collected and put in ultraconcentrator. Thrombin was added and the PRP was ready for use.	ABG iliac PRP(*n* = 32) ABG iliac (*n* = 27)	24/35	12	Radiograph

14. Imagama et al. [2]	Prospective	Lumbar PLF	7.74	400 mL of WB put in centrifuge for 15 minutes, buffy coat collected. Buffy coat put in centrifuge again for 15 minutes followed by the addition of thrombin.	ABG local bone and PRP(*n* = 29) ABG local bone (*n* = 29)	11/18	120	CT

CA = cage; PRP = platelet-rich plasma; ABG = autogenous bone graft; CT = computed tomography; IF = interbody fusion; PLF = posterolateral fusion; TLIF = transforaminal lumbar interbody fusion; PVB = peripheral venous blood; WB = whole blood; PLT = platelet; Conc = concentration.
